# The Role of Amaranth, Quinoa, and Millets for the Development of Healthy, Sustainable Food Products—A *Concise* Review

**DOI:** 10.3390/foods11162442

**Published:** 2022-08-13

**Authors:** Gayathri Balakrishnan, Renée Goodrich Schneider

**Affiliations:** Department of Food Science and Human Nutrition, University of Florida, Gainesville, FL 32611, USA

**Keywords:** cereals, ancient grains, bioactive peptides, plant-based food, dairy-free, sustainable, food security, health, protein, gluten-free

## Abstract

The selection of sustainable crops adaptable to the rapidly changing environment, which also cater to the dietary needs of the growing population, is a primary challenge in meeting food security. Grains from ancient crops such as amaranth, quinoa, and millets are positioned to address this challenge and hence have gained dietary predominance among cereals and pseudocereals due to their nutritional value and energy efficiency. From a nutritional perspective, they are recognized for their complete protein, phenolic compounds and flavonoids, prebiotic fibers, and essential micronutrients, including minerals and vitamins. Bioactive peptides from their proteins have shown antihypertensive, antidiabetic, antioxidant, and anticancer properties. The nutritional diversity of these grains makes them a preferred choice over traditional cereals for developing healthy, sustainable food products such as plant-based dairy, vegan meats, and gluten-free products. With growing consumer awareness about sustainability and health, the categories mentioned above are transitioning from ‘emerging’ to ‘mainstream’; however, there is still a significant need to include such healthy grains to fulfill the nutritional gap. This review article emphasizes the health benefits of amaranth, quinoa, and millet grains and discusses the recent research progress in understanding their application in new sustainable food categories. The challenges associated with their incorporation into novel foods and future research directions are also provided.

## 1. Introduction

Food security is defined by the UN Committee of World Food Security as ‘all people, at all times, have physical, social, and economic access to sufficient, safe, and nutritious food that meets their food preferences and dietary needs for an active and healthy life’ [1]. The growing world population, climate change, and rising demand for agricultural commodities have created invincible challenges in achieving this status globally. Cereal products have been a staple food in the human diet since prehistoric times; among cereal crops, corn, wheat, and rice are the most widely grown and consumed products worldwide [2]. To keep pace with the growing population, current agricultural practices have focused on maximizing the production of these three crops with modern technology and research advancements, particularly hybrid breeding programs, chemicals to control diseases, fertilizers, and modern irrigation systems [3]. However, such approaches pose two primary concerns to food security: (a) Cultivation of these crops using the above-mentioned extensive methods has led to the increased emission of greenhouse gases resulting in the pollution of natural resources. Their production is also heavily reliant on natural resources that are becoming scarce due to climate change. In developing countries such as India and other regions of Asia, where freshwater accessibility is already declining, the production of such crops consumes 70% of the water available for agriculture [4]. Rapid climate change has led to a significant increase in temperature and prevalence of drought conditions, which have limited the availability of arable land for crop cultivation (b) Limiting the dietary intake to food products developed from rice, corn, and wheat grains has shown insufficiency in meeting nutritional requirements leading to malnourishment in underdeveloped and developing countries [5]. The protein quality of wheat, corn, and rice is considered poor because of one or more limiting amino acids, such as tryptophan and lysine [6]. According to the FAO report ‘The State of Food Security and Nutrition in the World 2021′, a staggering three billion people suffered from malnourishment and did not have access to healthy diets in 2019.

**Figure 1:**
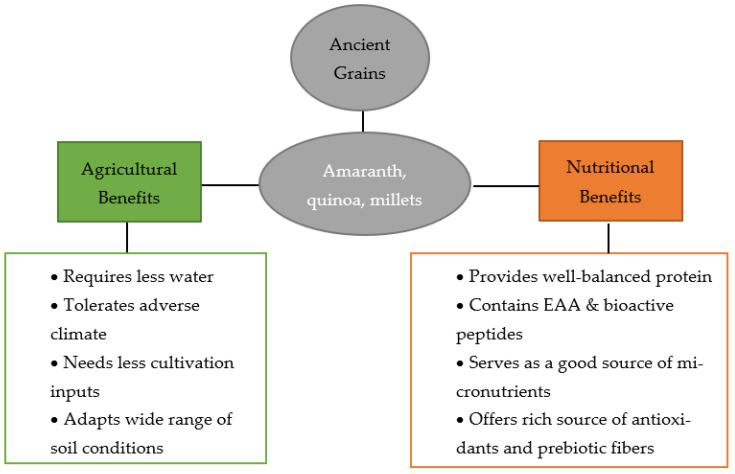
Agricultural and nutritional benefits of amaranth, quinoa, and millets.

To overcome this dire situation, it is pivotal to shift to crop diversification by including crops that are environmental-friendly (i.e., crops that are tolerant of harsh conditions and need less input of the agricultural resources) and would help curb malnourishment and micronutrient deficiency among the vulnerable population. Considering the above two aspects, the category of ancient grains offers a plethora of benefits owing to their rich chemical composition, such as high-quality protein, micronutrients, and various bioactive compounds, along with their adaptiveness to extreme climatic/soil conditions, such as drought, salinity, frost, and dry/arid regions (Figure 1). While there is no official definition for ancient grains, the term refers to cereal-like crops that have remained genetically unchanged over several hundred years [7]. In recent years, the grains from this category that have sparked consumer and research interest are amaranth, quinoa, and millets.

Amaranth and quinoa are native to the Andean region, belonging to the Amaranthaceae family. Both crops share similarities in their historical background and botanical identity. Amaranth and quinoa seeds have been consumed in Central and South America for over 5000 years and were an essential component of the diet of ancient cultures such as the Aztec, Mayan, and Incan civilizations [8,9]. Quinoa was referred as the ‘mother grain’ because of its exceptional nutritional value [10], and it was regarded as a gift from their gods by the Inca people. Although they were a staple food along with maize and potatoes, their production and consumption were suppressed after the Spanish conquest, and thereafter their cultivation was limited to a much smaller scale [11].

Botanically, amaranth and quinoa seeds are called pseudo-cereals as they are dicotyledonous, unlike true cereals that are monocots. The seed structure varies from traditional cereals and has three distinct layers: perisperm, embryo, and seed coat. The perisperm forms the center of the seed and is the central storage reserve for the developing embryo [12]. The embryo wraps around the perisperm, which, in turn, is encapsulated by a membranous layer called the testa or seed coat [13]. Amaranth seeds are lenticular and small (0.9–1.7 mm diameter) with a weight ranging from 0.6 g to 1 g for 1000 seeds [14]. The seed coat varies in color from white, gold, brown, pink, and black. While several species of amaranth are grown for their seeds and leaves, the three species commonly cultivated for their seeds are *A. caudatus, A. cruentus,* and *A. hypochondriacus.* Similar to amaranth, quinoa (*Chenopodium quinoa*) is an annual plant, 1–2 m tall with deep penetrating roots, that produces tiny, round, flattened seeds that measure 1.5 mm in diameter, with an approximate weight of 1 g for 350 seeds [12]. Quinoa seeds vary in color and size; the common seed varieties available on the market are pale yellow, red, and black. Until the past decade, the growth of these crops was confined to Central and South America. However, their cultivation is under investigation in other parts of the world, such as the USA Canada, and several countries in Europe.

Millets are another class of ancient crops that produce grains, with a promising potential to curb food insecurity. Millets belong to the grass family Poaceae (or Gramineae) grown in the semi-arid tropics of Asia and Africa [15]. The term millets broadly refer to a heterogeneous group of crops known for their small coarse grains and they are classified into great/large millets and small millets depending on their grain size. Finger millet (*Eleusine coracana*), proso millet (*Panicum miliaceum*), foxtail millet (*Setaria italica*), and kodo millet (*Paspalum scrobiculatum*) are some of the small millet varieties, while sorghum (*Sorghum bicolor*) and pearl millet (*Pennisetum glaucum*) belong to the great/large millets [16]. Historically, small millets were indigenous to South Asia and served as a predominant crop for the Indus civilization. There are differences in production requirements between large and small millets; compared to large grain millets, small millets grow well in various soils and climatic conditions, require less energy for growth, and need much less management overall [17]. Pearl millet is the primary type of millet harvested globally, accounting for 46%, followed by foxtail, proso, and finger millet [18]. The grain morphology of millets is similar to other cereal grains with three distinct layers, namely the endosperm, germ, and an outer bran layer. Compared with other major cereals, millets remain an underutilized crop, though there has been increased interest in their utilization in recent years. This review aims to highlight the nutritional superiority of these selected seeds and grains, particularly the benefits of their protein and peptides, along with the application for developing healthy sustainability-based food products that are important for consumers.

## 2. Nutritional Significance and Health Benefits of Ancient Grain Proteins

The most important benefit of ancient grains that have led to the recognition of their nutritional value is protein quality. The nutritional quality of protein is driven by its ability to provide essential amino acids that cannot be synthesized by our human body and thus needs to be obtained through our diet. The concentration and ratios of amino acids present in a specific protein are paramount in understanding its nutritional quality—the greater the ratio of essential amino acids, the greater the protein quality [6]. Along with the amino acid composition, protein digestibility and absorption also play crucial roles in determining the nutritional quality of a particular product. Typically, animal-derived proteins such as milk and eggs are considered ‘complete’ because they contain the nine essential amino acids and have a high protein digestibility score. Conversely, most cereal proteins are considered inferior due to inadequate levels of essential amino acids and poor digestibility. Table 1 lists the total protein and the concentration of each amino acid in these ancient grains. All three grains of interest have high quality protein comparable to milk and egg, with essential amino acids that are not present in traditional cereals. The overall seed/grain protein content of amaranth, quinoa, millets are in the range of 13.4–16.5%, 12.1–14.5%, and 7–11%, respectively, approximately twice the amount present in wheat, maize, and rice [19,20]. Compared to wheat grain, quinoa has twice the amount of lysine, tryptophan, and methionine [21,22]. A study by Nimbalkar et al. [23] found amaranth seeds to be high in lysine and sulfur-containing amino acids as opposed to wheat that is only rich in non-essential amino acids such as glycine and proline. Aside from the rich amino acid composition, the protein digestibility of amaranth (79–89%) and quinoa seeds (92%) were comparable to the milk protein casein (100%) [22,24]. Because of the remarkable protein content and amino acid profile, NASA classified quinoa as an emerging crop with excellent nutritional properties for long-term human space missions. Unlike amaranth and quinoa, millets are deficient in lysine, but they are rich in vital amino acids such as methionine and cysteine that are absent in traditional cereals [25]. When comparing proso, foxtail, and finger millets, Ravindran [26] noted that finger millet had a well-balanced amino acid composition with higher amounts of lysine, threonine, and valine than the other two millets. In the same study, the protein digestibility of raw millets ranged between 70 and 78%, but it was found to improve significantly (88–92%) after cooking. The author noted that the increase in protein digestibility was due to the inactivation of heat-sensitive antiproteinase factors that were present in the raw grains. Although raw grains are seldom consumed due to their low palatability; the use of cooking/processing is also necessary to enhance their protein utilization 

**Figure 2:**
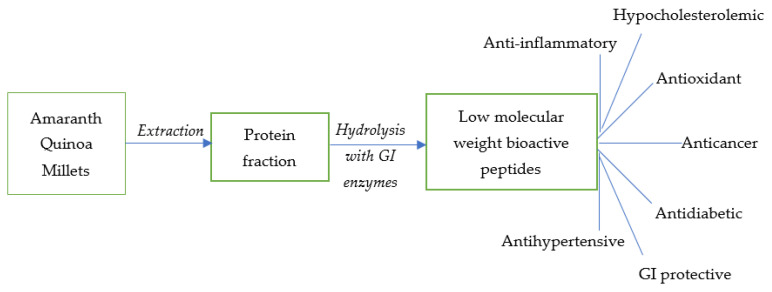
Health benefits of bioactive peptides extracted from amaranth, quinoa, and millet proteins.

Besides the exceptional protein quality and quantity, bioactive peptides from ancient grain proteins have been shown to possess several key health benefits. Bioactive peptides are a mixture of free amino acids and low molecular weight peptides of varying chain length (2–20 amino acid residues) released from the proteins due to the action of gastrointestinal enzymes under physiological conditions. Smaller peptides are released from proteins due to the action of digestive enzymes such as pepsin, trypsin, chymotrypsin, and peptidases. Bioactive peptides have the tendency to regulate important physiological processes, and have different biological activities including antioxidant, antihypertensive, antidiabetic, antimicrobial, and hypocholesterolemic effects (Figure 2). Although assessment of the release of these compounds under in vivo conditions is complex, several studies have investigated the health benefits of peptides derived from ancient grains under in vitro conditions after treatment with gastric and intestinal enzymes (Table 2). To generate bioactive peptides under gastrointestinal conditions using an in vitro model, the gastric and intestinal phases are simulated using pepsin at pH 2.0 for 2–3 h, followed sequentially by pancreatin at a higher pH of 7.0–7.5 for 3–4 h, respectively. One such study by Vilcacundo et al. [31] showed peptides released from quinoa seeds had a free radical scavenging capacity and the ability to inhibit tumor growth in various colon cancer cell lines such as Caco-2, HT-29, and HTC-116. Smaller peptide fractions <5 kDa had antioxidant activity while larger peptides >5 kDa were found to be responsible for anticancer activity. The same author also demonstrated that the antidiabetic effects of quinoa peptides were due to enzyme inhibition of α- amylase, α-glucosidase, and dipeptidyl peptidase IV. Guo et al. [32] showed that quinoa peptides obtained through in vitro digestion exhibited antihypertensive effects in rats in the form of ACE inhibitory activity. Similar to quinoa, the peptides from raw and heat-treated amaranth seeds showed anticancer activity by apoptosis and cell growth inhibition when evaluated using breast cancer cell lines [33] and HT-29 colon cancer cells [34]. Jakubcyzk et al. [35] reported glycine-rich peptides released from proso millet grains, after hydrolysis by gastrointestinal enzymes, had anti-inflammatory effects against lipoxygenase and al. [36] proved that protein hydrolysates from foxtail millets improved the integrity of the intestinal barrier and reduced the expression of pro-inflammatory cytokines that cause inflammation of the intestine in irritable bowel disease. An in-depth investigation carried out by Agrawal et al. [37] showed that peptides <3 kDa produced by sequential proteolysis of finger millet proteins by pepsin and trypsin had significant antioxidant activity. The authors identified the presence of hydrophobic amino acids that stabilized free radicals through hydrogen interactions with target molecules such as DPPH and ABTS. 

All the above-mentioned research shows the similarity in the health benefits associated with ancient grain proteins and bioactive peptides. Their superior protein quality makes them an ideal ingredient for enhancing the nutritional adequacy of specialty products developed for environmental- and health-conscious consumers. The following sections discuss the recent studies that have investigated the utilization of ancient grains in such emerging, novel food products, namely plant-based food substitutes and gluten-free products that often rely on these grains to boost their nutritional quality.

## 3. Application of Ancient Grains in Emerging Sustainable Based Food Applications

### 3.1. Plant-Based (Dairy-Free) Beverages

A growing number of consumers choose plant-based milk/beverage alternatives for reasons related to health, such as lactose intolerance and allergies to cow’s milk, which is prevalent among 80% of the population worldwide [35]. In addition, since the dairy sector is one of the largest contributors of greenhouse gas emissions, consumers seek plant-based products to adopt sustainable diet practices (e.g., vegan) and reduce the negative environmental impact [44]. Since current plant-based milk in the market has much less protein (<1% *w*/*v*) than cow’s milk [45], emerging research on the extraction of milk/beverages using ancient grains has been shown to deliver a protein content that nutritionally matches dairy beverages. Pineli et al. [35] investigated the development of quinoa milk by adapting the same process used for rice milk extraction. Their study showed quinoa milk had 5.66-fold higher protein than rice milk and half the protein content of cow’s milk. The reported protein content of quinoa, rice, and cow’s milk were 1.7%, 0.3%, and 3.2%, respectively. The lipids and sodium were significantly lower in quinoa milk than in cow’s milk. A similar investigation by Mäkinen et al. [46] yielded similar results for protein obtained from quinoa milk. However, these studies reported a low sensory acceptance of quinoa milk arising from its strong nutty aroma and flavor. Huang et al. [47] evaluated the production of quinoa milk using extrusion cooking and oat beta-glucan as a stabilizer. In contrast to previous studies, the authors found a high overall liking from consumers because of the extrusion process and higher viscosity from oat beta-glucan. A recent study by Manassero et al. [45] reported a high-protein low-calorie amaranth beverage with a protein content equivalent to cow’s milk and a calcium content higher than other plant-based milks; Although the nutritional significance of the amaranth milk was far better than existing plant-based milks, the sensory acceptability of the product is yet to be understood as the study did not look into consumer acceptance of the final product. Another recent study by Bembem and Agrahar-Murugkar [48] developed a ready-to-drink sorghum and finger-millet-based beverage, sweetened naturally with jaggery. The final product had a protein content of 11 g/100 g with consumer acceptability scores over 7.0 on a 9-point hedonic scale. The above studies have shown the possibility of successfully matching the nutritional value of plant-based milk to cow’s milk using ancient grains. However, most research has also reported palatability and consumer acceptability challenges often associated with these grains. Notably, the final product was associated with unpleasant flavors, and a diluted chalky texture and mouthfeel [47], which are major sensory hurdles to be addressed for the commercial success of these products in the market.

One of the ways to overcome the sensory challenge is fermentation, which changes the earthy and raw notes of cereal grains into a more dairy-like and pleasant sourness [49]. Fermentation using lactic acid bacteria (LAB) results in probiotic dairy products promoting gut health, improving protein digestibility and nutrient bioaccessibility, and reducing anti-nutritional factors [50,51]. Such fermented beverages from ancient grains have long been part of Asian and African food cultures. However, recently, there has been a more significant effort to transition them from traditional home-based products to commercial value-added goods [52]. Isaac-Bamgboye et al. [53] compared an indigenous Nigerian beverage called ‘kunu’, prepared traditionally from sorghum, against amaranth kunu. The study reported higher protein, and lower antinutrients such as oxalates, tannins, and saponins from amaranth kunu than sorghum. Although their preliminary sensory evaluation had only ten panelists, the authors reported better acceptability of amaranth kunu over sorghum. Urquizo et al. [54] developed a fermented quinoa beverage from quinoa flour, fermented with *Lactobacillus plantarum* Q823. Their study obtained a beverage with viable microbes that were stable for up to 28 days of storage (10^9^ CFU/mL). Although the unflavored product in their study was disliked due to its sour taste, the chocolate and bilberry flavors were found to have positive feedback. A similar study by Lorusso et al. [50] investigated the fermentation of quinoa using three different strains of lactic acid bacteria for developing a yogurt-like beverage. The study found an increase in essential amino acids and protein digestibility by 10–20% following a 20 h fermentation; a trained sensory evaluation of the yogurt-like products showed low scores for the earthy, cereal, artificial notes that were negatively associated with the product. Ziarno et al. [55] analyzed the properties of a fermented dairy-free millet beverage prepared using *Lactobacillus delbrueckii* subsp. *bulgaricus* and *Streptococcus thermophilus*. The finished product was free of dairy allergens such as milk protein and lactose, with sensory characteristics acceptable to vegetarian and lactose-free consumers. The research studies mentioned above have shown the value of fermentation for developing a probiotic beverage using ancient grains with improved sensory characteristics. However, further research and process investigations are needed to optimize the taste and texture and develop different flavor strategies that would offset the cereal taste, which negatively impacts consumer liking of these products.

### 3.2. Gluten-Free Applications

Wheat is the most common cereal containing gluten protein. Avoiding gluten is critical for consumers who have celiac disease (CD) since the only effective treatment for these individuals is the elimination of gluten from their diet. Celiac disease refers to the abnormal response of the body’s immune system to the gluten protein found in wheat, rye, barley, and oats. This condition damages the lining of the small intestine and reduces the absorption of nutrients (iron, calcium, vitamins A, D, E, K, and folate) [56]. Aside from CD individuals, consumers who experience gluten sensitivity and allergy also rely on gluten-free products to fulfill their dietary needs. Similar to plant-based dairy alternatives, a segment of consumers also chooses to follow a gluten-free diet for sustainability reasons because of the water consumption, and pesticide and fertilizer application involved in the cultivation of wheat. The current gluten-free products on the market are made using refined flour such as rice, which often lacks essential nutrients. Similar to rice, ancient grains do not contain the prolamin storage proteins that cause CD; hence the interest in exploring their application for gluten-free products has increased to enhance the nutritional richness of these products. Several processing challenges need to be addressed to incorporate ancient grains into GF products successfully. Gluten protein is an essential component that provides viscoelasticity and structure to wheat-based products such as pasta, bread, biscuits, and cookies. The absence of gluten impacts multiple sensory dimensions of a product, including the texture, appearance, and taste, leading to a negative consumer perception. Cairano et al. [57] carried out a comprehensive investigation of the nutritional and functional properties of gluten-free flours from 12 different sources of cereals and legumes. In their study, flours from millet, quinoa, and amaranth had a lower predicted glycemic index (pGI) than rice flour, a positive indicator for slower starch hydrolysis and release of sugars that spike blood glucose after consumption. Among the three ancient grains, millets were found to have a higher amount of resistant starch and lower pGI than amaranth and quinoa flour. From a functional perspective, quinoa, millet, and amaranth had a water absorption capacity (WAC) and oil absorption capacity (OAC) comparable to rice flour. These properties are desirable for gluten-free baked goods since a higher WAC and OAC are necessary for a softer texture, improved mouthfeel, and flavor retention in finished products. Along with ancient grains, additional ingredients such as hydrocolloids, enzymes, starches, and emulsifiers are routinely used to improve the processing stability in the absence of gluten. The below case studies emphasize the recent research that has investigated the effect of one or more ancient grains for the preparation of gluten-free products along with the use of the additives mentioned earlier.

### 3.3. Gluten-Free Baked Goods

A study by Moss and McSweeney [58] examined the sensory and consumer acceptability of GF bread prepared using chia seed, quinoa, and millet, compared to those prepared using brown rice flour. Their study indicated that the use of millet at lower proportions (25% of the formulation) led to a consumer liking comparable to the bread from brown rice flour. The same study also found that the quinoa bread had a nutty, bitter, astringent flavor profile, leading to less consumer liking than brown rice flour bread. Another study by Drub et al. [59] evaluated the complete replacement of rice flour and starch mixtures in the yeast-leavened GF bread with millet, amaranth, quinoa, buckwheat, brown rice, or sorghum flour. Of the several grains that were investigated, millet bread yielded the best results with a loaf volume comparable to the control, superior protein and fiber content, and sensory acceptability scores on par with the control. A 100% replacement with quinoa or amaranth flour improved the nutritional quality of the bread with a similar sensory acceptance to the control bread. However, the study noted a lower loaf volume due to interference of fiber/protein with the starch gelatinization. Both of these two studies used xanthan gum that functioned as a dough strengthener and improver. Sarabhai et al. [60] researched the dough and bread properties of the foxtail millet bread formulated using protease, glucose oxidase, and xylanase. The enzyme-treated dough provided a finished product with a higher bread loaf volume and uniform crust formation compared to the bread without enzymes. Of the three enzymes, protease had the highest sensory acceptability due to its improved viscosity and batter development resulting from protein hydrolysis. A novel approach investigated by Nami et al. [61] involved using *Lactobacillus* cultures for the bread fermentation prepared from pearl millet flour. *Lactobacillus* fermentation (sourdough) promoted the leavening process by trapping carbon dioxide, leading to an enhanced crumb texture and flavor of the bread. Their study concluded that sourdough fermentation improved the loaf volume and crumb softness and prolonged the bread’s shelf life. 

Besides bread, the gluten-free cookie is another product category where product optimization is critical for the proper delivery of flavor and texture to match the sensory characteristics of wheat-based cookies. Jan et al. [62] optimized the process parameters of producing gluten-free cookies using quinoa flour. The authors optimized the fat, sugar, baking temperature, and baking time of quinoa flour cookies and produced a final product with a consumer acceptability score above 7.0 on a 9.0-point scale. Sharma et al. [63] compared cookies formulated using a germinated vs. ungerminated millet blend (foxtail, barnyard, and kodo millet); their study found cookies formulated at a ratio of 70:20:10 had a sensory liking comparable to wheat cookies and were significantly higher in protein, dietary fiber, and antioxidants.

### 3.4. Gluten-Free Pasta

Gluten-free pasta has presented substantial formulation challenges for researchers since gluten forms the backbone of conventional pasta structure. The replacement of wheat flour with gluten-free flours leads to a poor texture characterized by stickiness, shape deformation, and significant cooking losses. The application of hydrocolloids for making gluten-free pasta has been extensively explored to eliminate the above concerns and improve the textural properties. Romera et al. [64] improved the dough stability and texture of proso millet flour to prepare spaghetti using hydrocolloids such as guar gum and xanthan gum. Their study noted that spaghetti made using millet without gums led to complete disintegration of the noodles while cooking, while the addition of hydrocolloids led to a stable product. However, the final spaghetti still had a less desirable texture than wheat-based pasta. Chauhan et al. [65] evaluated amaranth flour with hydrocolloids guar gum, gum acacia, and gum tragacanth, each added at 0.5% and 1.0% of the formulation. Although their study found significant differences between amaranth pasta and semolina pasta for color, texture, cooking, and sensory characteristics, their study showed that guar gum at a 1% usage level performed as a promising hydrocolloid to improve the overall pasta quality, along with delivering 15% protein. Rudra et al. [66] found that the extrusion processing improved pasta firmness made using a combination of millet and amaranth flour. The authors also noted that gluten-free pasta had a much higher ease of protein digestion than semolina (wheat-based) pasta, which would benefit celiac consumers.

While the use of ancient grains on their own may not yield desirable gluten-free products sensorially, the inclusion of additional ingredients provides promising results in meeting the taste and texture expectations of consumers, along with boosting the nutritional quality of these products.

### 3.5. Plant-Based Meat Alternatives

Another emerging area in applying ancient grains is in vegan meat alternatives that employ plant-based proteins to address the protein needs of flexitarians (people who reduce meat due to health/environmental reasons) and vegetarian/vegan consumers. The current plant-based meat products on the market heavily rely on soybean and wheat gluten, which can be allergenic to potential consumers. Although there is minimal research evidence in the use of ancient grains so far, the existing literature provides an opportunity to investigate ancient grains for the full-scale production of meat mimics that will be beneficial for creating meat-free products. Recent research by Felix et al. [67] isolated and characterized quinoa flour gels; their study showed that quinoa gel formed a sausage-like texture due to the synergetic effect of carbohydrates and proteins. The strength of the quinoa gel was dependent on both the quinoa flour concentration and gelation temperature, with the latter having a higher impact on gel formation. The study noted that proper formation of quinoa gels occurred when the processing temperature was above the starch gelation temperature (65 °C), where there was breakdown of amylose and amylopectin that strengthened the gel structure. Although the study did not evaluate the use of quinoa gel in vegan-based products, its successful isolation and characterization show its applicability for meat alternatives.

Moreover, quinoa protein isolates have similar foaming and emulsification properties as the soy protein commonly used today in vegan meat applications. A study by Das et al. [68] aimed to understand the effect of the extraction pH on the functional and rheological characteristics of amaranth protein isolate. The gelation values (ability to form an elastic gel network) were higher for amaranth protein extracted at pH 9.0 than other pHs included in the study, which is an essential functional aspect that affects the texture and mouthfeel of meat-free applications. The research in this area is at nascent stages; hence, exploration of the functional suitability of ancient grain proteins for plant-based meat substitutions is much needed. Further exploration of ancient grains will be hugely beneficial to developing new products in this rapidly growing category to meet the protein demand without raising allergen concerns associated with current ingredients such as soy protein and wheat gluten.

## 4. Nutritional Benefits of Ancient Grains—Beyond Proteins

Aside from proteins, ancient grains have higher amounts of minerals than conventional cereals, which are crucial for tackling micronutrient deficiencies worldwide. Notably, ancient grains are rich in calcium, iron, zinc, and potassium that are not present in staples such as wheat, rice, and maize (Table 3). Since these minerals are vital for cognitive development and physical functions, diversity in the diet with the incorporation of such grains is important to meet mineral requirements. Additionally, processing before consumption is also necessary for mineral absorption since ancient grains contain inhibitors such as phytates and tannins that interfere with the bioavailability of minerals. The type of interfering anti-nutritional elements and the application of roasting, cooking, germination, and fermentation to reduce their content has been discussed elsewhere in the literature [69,70,71,72].

Ancient grains are also a rich source of phenolic compounds that have shown antioxidant and anti-inflammatory properties in vitro and in vivo animal models. The key compounds identified in these grains include flavonoid glycosides such as quercetin, kaempferol, phenolic acids, tocopherols, carotenoids, and tannins [76,77,78,79]. Furthermore, these grains also have dietary fibers that are prebiotics promoting the presence of beneficial microbes in the intestine, leading to enhanced gut health. Zeyneb et al. [80] showed that quinoa polysaccharides increased *Bifidobacterium* and *Collinsella* after in vitro digestion. Similarly, amaranth seeds and millets have xyloglucans- and arabinose-rich polysaccharides that have been shown to increase the concentration of *Lactobacillus* and *Bifidobacterium* in the large intestine [81]. *Bifidobacterium*, *Lactobacillus*, and *Collinsella* are important gut microbes with beneficial physiological functions such as enhancing immunity, balancing gut microbiota, defending against pathogens, and improving overall health.

## 5. Clinical Trial Evidence on the Consumption of Amaranth, Quinoa, and Millets

The health benefits of various bioactive substances derived from ancient grains are well-established in the literature using in vitro chemical and biological assays. Although the results have been promising, it is pivotal to evaluate such health claims in vivo using human clinical studies. A handful of recent studies have documented the effectiveness of consuming the three ancient grains against chronic disease conditions. An early study by Chávez-Jáuregui et al. [82] evaluated the consumption of an extruded amaranth snack on the lipid profile of patients suffering from hypercholesterolemia. Although the study found a reduction in LDL, VLDL, total cholesterol, and triglycerides, none of the parameters were significantly different compared to the placebo group. The reasons for the lack of significant difference acknowledged in the study were the small sample size and product quantity. Hence, a dose-dependent study with more subjects will help increase the data quality of such studies.

A study by Navarro-Perez et al. [83] investigated the dose-dependent effect of quinoa seeds in lowering serum triglycerides among overweight and obese adults. The team measured the lipid profile, body composition, and nutrient intake of the adults consuming quinoa seeds (25 g or 50 g/day) vs. control for 12 weeks. The study found a significant reduction in serum triglycerides among participants who consumed 50 g quinoa seeds/day, although other biomarkers such as total cholesterol, HDL, and LDL were unchanged. Higher serum triglycerides are often a risk factor for cardiovascular disease (CVD), and hence the 36% reduction authors noted in the study is a positive indication of the potential of consuming quinoa to reduce the risk of CVD. A meta-analysis performed by Atefi et al. [84] also suggested supplementation of quinoa at doses above 50 g could lower the risk of CVD by reducing serum triglycerides in adults. Another randomized, cross-over study observed the effects of quinoa biscuits on CVD biomarkers for four weeks. The study was carried out on healthy older adults in which the researchers found a decrease in total and LDL cholesterol, body weight, and BMI in the quinoa group compared to the control group. Although the authors hypothesized the presence of polyunsaturated fatty acids as the reason behind the observed lipid-lowering property, the outcome did not support their hypothesis; the amount of PUFA fatty acids was unchanged in the participants after quinoa consumption [85]. Although the above studies have shown moderate changes in biomarkers related to CVD, the mechanism behind such an effect is yet to be understood. 

Ren et al. [86] examined the antidiabetic effects of the foxtail millet diet in subjects with impaired glucose tolerance. The study was carried out for 12 weeks, where the participants consumed foxtail millet bread equivalent to 50 g/day of whole grains. The study showed a significant reduction in fasting blood glucose and 2-h glucose after meals between the beginning and the end of the 12 weeks. Leptin, an important hormone that suppresses hunger and maintains energy balance, was also higher after consuming foxtail millet for 12 weeks. A similar improvement in the glucose levels of diabetic individuals was noted after the intake of finger millet buns for 60 days. In the same study, other metabolic parameters such as serum cholesterol, triglycerides, LDL, and HDL also showed modest changes after continuous consumption of finger millet [87]. Besides diabetes, foxtail millet has also reduced blood pressure in mildly hypertensive adults [88]. The study noted a significant reduction in systolic and diastolic blood pressure at the end of 12 weeks of intervention. The anti-hypertensive effect of the millet was linked to ACE (angiotensin converting enzyme) inhibition in the serum, which was seen as a downward trend (not statistically significant) after 12 weeks.

Table 4 provides a list of clinical trials carried out thus far to evaluate the benefits of ancient grains using various biomarkers in human subjects. Most of the research has measured glucose, lipid profile, and inflammatory markers that emphasize the understanding of the antidiabetic, anti-obesity, anticholesterolemic, and anti-inflammatory potential of these grains. However, there are a few critical shortcomings in conducting such clinical trials that need to be addressed: (1) The intervention period and dosage of the grain vary broadly from study to study. Many trials were conducted for a short 4–8 week duration, which may not provide sufficient data to indicate the benefits from these grains obtained over a prolonged period adopted as part of the everyday diet. (2) The number of trial participants should be statistically determined using a power analysis. Several previously held studies did not find significant treatment effects, partly because the study was underpowered due to a very small sample size. (3) While the benefits of amaranth, quinoa, and millets are evident from the clinical trials, the underlying mechanism behind the observed health benefits is largely unknown.

## 6. Summary and Future Research

Food insecurity is a global concern regarding the lack of nutritious food choices. It is a multifactorial issue impacted by economic, agricultural, environmental, and social changes. Climate change, in particular, is a severe threat to food security because of its substantial impact on the agricultural sector. The progressive increase in global temperature and the decline in available water and cultivable land are some of the critical factors causing a decrease in crop yield. Any such negative impact on the agricultural industry disrupts the food supply chain and access to nutritious food. Although agricultural advancements have found solutions to maximize the production of staple crops, the transformation to utilize sustainable crops with a low environmental impact is necessary to overcome the adverse effects of climate change. Since consumption of staple crops alone often leads to the lack of essential nutrients, diet diversity is vital for preventing malnourishment.

Hence, to fight food insecurity, it is of utmost importance to develop sustainable crops resilient to climate change and formulate food products that deliver key nutrients for maintaining a wholesome diet. Amaranth, quinoa, and millet grains, discussed in this review, fulfill both these criteria, and would help to contribute to food security. The crops possess physiological characteristics that make them resistant to drought, high salinity soils, and high temperature; they also thrive well in low-fertility soils. Although these crops have been a part of the culture in developing countries in Asian, South, and Central American regions, the lack of awareness of their benefits has limited their consumption in these countries. Educating farmers about the possibility of growing these grains without a more significant need for natural resources would lead to faster adoption of these crops by the agricultural community.

It is also equally important to raise awareness among consumers about the health benefits of consuming these grains. The lack of information on their nutritional profile and preparation techniques has reduced their consumption over the years. Even within the food industry, there is still a significant knowledge gap in utilizing ancient grains to develop scalable food products. Future research should devote resources to identifying process strategies that will lead to the creation of novel products that are commercially viable. Notably, further research is needed to find the right solutions for the challenges associated with including these grains in sustainable product categories such as plant-based diets, highlighted in this review. Additional research is also needed to optimize formulations that would influence their consumer appeal without losing their nutritional quality.

Malnutrition is a primary concern for food insecurity; the presence of high-quality proteins, minerals, vitamins, and other bioactive substances with benefits against biomarkers of various diseases such as diabetes, cancer, and hypertension would contribute to addressing nutritional inadequacy. While the health benefits have been well-established in the literature using in vitro models, there is a need to investigate them using clinical trials to understand their efficacy under human physiological conditions. The clinical trials conducted so far with the three grains have had limited scope due to fewer participants involved in the research. In-depth research with various biomarkers and a statistically reliable sample size is needed to further understand the health benefits and underlying mechanisms. Aside from proteins and micronutrients, ancient grains also have unique phenolic compounds, and prebiotic/probiotic benefits that often may not be present in traditional staple cereals. Research regarding antioxidants and prebiotics from these grains is scant; further focus on exploring these areas will be valuable because of their role in preventing disease.

In summary, the nutritional advantage and resilience to extreme climates position the ancient grains amaranth, quinoa, and millets to become a part of the solution to end food insecurity in developing and underdeveloped countries where they are predominantly grown. Channeling the research efforts on the production and process strategies, clinical assays, and the creation of differentiated affordable products with sensory appeal would lead to the successful integration of these grains into the daily diet of consumers.

## Figures and Tables

**Table 1:** Protein and amino acid profile of ancient grains.

Ancient Grain	Amaranth	Quinoa	Finger Millet	Foxtail Millet	Proso Millet	FAO/WHO Amino Acid Reference Pattern for Adults (g/100 g Protein)
Protein (g/100 g)	13.6	14.1	9.8	15.9	14.4	
Essential amino acids (g/100 g protein)						
Histidine	3.0	2.7	2.8	2.3	2.4	1.5
Isoleucine	3.9	3.1	5.2	5.1	4.9	3.0
Leucine	6.2	6	11.7	16.0	14.0	5.9
Lysine	5.7	4.8	3.1	1.9	1.7	4.5
Methionine+ Cystine	4.6	3.3	5.9	5.1	5.1	2.2
Phenylalanine + Tyrosine	5.4	6.3	10.3	10.0	10.8	3.8
Threonine	5.1	3.7	5.2	4.5	4.1	2.3
Tryptophan	0.9	0.9	1.3	1.1	0.6	0.6
Valine	5.9	3.7	8.2	6.3	6.4	3.9

Adapted from: Ravindran, et al. [26], Malleshi et al. [27], Parameshwaran et al. [28], Nowak et al. [29], and Saguro Nieto et al. [30].

**Table 2:** Summary of recent studies that assessed the health benefits of ancient grain bioactive peptides after in vitro gastrointestinal digestion.

Ancient Grain	Health Benefits	Research Finding	References
Amaranth	Antioxidant, anti- inflammatory properties	Germinated amaranth released bioactive peptides that reduced nitric oxide production and showed antioxidant effects	[38]
	Anti-cancer	Antiproliferation, induction of apoptosis, and necrosis of cancer cells	[39]
	Antioxidant	Intracellular reduction of the reactive oxygen species	[34]
	Antioxidant and anti-cancer	Radical scavenging activity, inhibition of cancel cell growth, DNA fragmentation leading to apoptosis	[40]
Quinoa	Antioxidant and anti-cancer properties	Seventeen peptides with antioxidant activity tested using ORAC, and inhibition of tumor cell viability in cancer cell lines	[33]
	Anti-hypertension	Low molecular peptides containing arginine, phenyalanine, and proline that inhibited ACE activity in vitro, and blood pressure in hypertensive rats	[31]
	Anti-cancer	Smaller peptides <5 kDa with antiproliferative activity modulated by HDAC1 inhibition	[32]
	Antidiabetic	Three peptides were identified from quinoa globulin proteins. Antidiabetic activity due to DPP-IV, α-amylase, and α-glucosidase inhibition.	[41]
Finger Millet	Antioxidant activity	Two peptides with aromatic and hydrophobic amino acids that stabilized free radicals	[42]
Foxtail millet	Anti-inflammatory	Peptides from germinated and heat-treated millets that were effective against biomarkers of inflammation and oxidative stress	[37]
	Protection against intestinal inflammation	Presence of peptides that effectively inhibited inflammatory factors such as NF-kB, interleukin-6	[43]
Proso millet	Anti-inflammatory and lipase inhibition	Glycine-rich peptides with lipoxygenase and cyclooxygenase inhibitory activity	[36]

**Table 3:** Mineral content of ancient and common grains.

Minerals (mg/100 g)	Amaranth	Quinoa	Finger Millet	Foxtail Millet	Proso Millet	Wheat	Rice	Corn
Copper	0.51	0.59	4	3	4	0.41	0.3	0.054
Manganese	1.51	1.95	5	26	19	4.07	2.84	0.163
Iron	9.62	5.46	5	19	20	3.6	1.5	0.52
Zinc	5.55	2.93	2	9	11	2.6	1.91	0.46
Magnesium	231	197	130	100	120	137	124	37
Calcium	165	44	240	30	30	34	10	2
Phosphorus	527	468	240	270	260	357	319	89
Potassium	530	664	570	400	370	363	265	270

Adapted from: Ravindran [73], Nascimento et al. [74], USDA [75].

**Table 4:** Clinical trials on amaranth, quinoa, and millet products to understand their antidiabetic, anti-obesity, anticholesterolemic, and anti-inflammatory potential.

Ancient Grain	Key Biomarkers Measured	Number of Participants	Intervention Period	Significant Differences vs. Control	Reference
Amaranth	Plasma lipids	22		No	[82]
	Diabetes markers (insulin, leptin, resistin, visfatin, PAI-1)	62	3 months	Yes	[89]
Quinoa	Lipid profile (total cholesterol, triglycerides, HDL, LDL))	50	12 weeks	Yes (triglycerides)	[83]
	Glucose			No	
	Hormones (leptin, adiponectin, insulin, C-peptide			No	
	Lipid profile (total cholesterol, triglycerides, HDL, LDL)	27	120 to 200 days	Yes	[90]
	Glucose			No	
	Lipid profile (total cholesterol, triglycerides, HDL)	40	4 weeks	Yes (total cholesterol and LDL)	[85]
	Serum antioxidants			No	
	Serum PUFA			No	
Finger millet	Plasma antioxidants	18	8 weeks	Yes	[91]
	Cholesterol			No	
	Glucose			No	
	Glucose	30	60 days	Yes	[87]
	Lipid profile (total cholesterol, triglycerides, HDL, LDL, VLDL)			Yes (except for triglycerides and VLDL)	
Foxtail millet	Glucose	64	12 weeks	Yes	[86]
	Lipid profile (total cholesterol, triglycerides, HDL, LDL)			Yes (HDL and LDL)	
	Cytokine (IL-6, TNF-α)			Yes	
	Hormones (leptin, adiponectin, GLP-1)			Yes (only for leptin)	
	Blood pressure			Yes (diastolic)	
	Blood pressure	45	12 weeks	Yes	[88]
	RAAS (renin-angiotensin-aldosterone system)			No	
	Lipid profile (total cholesterol, triglycerides, HDL, LDL)			No	
	Fasting glucose			Yes	
Barnyard millet	Glucose	15 (9 diabetic and 6 non-diabetic)	28 days	Yes (only among diabetic participants)	[92]
	Lipid profile				
Millets (foxtail, sorghum, and finger millet)	Glucose	150	NA	Yes	[93]
	Lipid profile				

## Data Availability

Not applicable.

## References

[1] FAO (2011). FAO in the 21st Century: Ensuring Food Security in a Changing World. http://www.fao.org/3/i2307e/i2307e.pdf.

[2] Zamaratskaia G., Gerhardt K., Wendin K. (2021). Biochemical characteristics and potential applications of ancient cereals-An underexploited opportunity for sustainable production and consumption. Trends Food Sci. Technol..

[3] Gregory P.J., George T.S. (2011). Feeding nine billion: The challenge to sustainable crop production. J. Exp. Bot..

[4] Senthilkumar K., Bindraban P., Thiyagarajan T., De Ridder N., Giller K. (2008). Modified rice cultivation in Tamil Nadu, India: Yield gains and farmers’ (lack of) acceptance. Agric. Syst..

[5] Wakeel A., Farooq M., Bashir K., Ozturk L. (2018). Micronutrient malnutrition and biofortification: Recent advances and future perspectives. Plant Micronutrient Use Efficiency.

[6] Friedman M. (1996). Nutritional value of proteins from different food sources. A review. J. Agric. Food Chem..

[7] Taylor J., Awika J. (2017). Gluten-Free Ancient Grains: Cereals, Pseudocereals, and Legumes: Sustainable, Nutritious, and Health-Promoting Foods for the 21st Century.

[8] Kozioł M.J. (1992). Chemical composition and nutritional evaluation of quinoa (*Chenopodium quinoa* Willd.). J. Food Compos. Anal..

[9] Maurya N.K., Arya P. (2018). Amaranthus grain nutritional benefits: A review. J. Pharmacogn. Phytochem..

[10] Taylor J., Parker M.L. (2002). Quinoa. Pseudocereals and Less Common Cereals.

[11] Berghofer E., Schoenlechner R. (2002). Grain Amaranth. Pseudocereals and Less Common Cereals.

[12] Varriano-Marston E., DeFrancisco A. (1984). Ultrastructure of quinoa fruit (*Chenopodium quinoa* Willd). Food Struct..

[13] Bruno M.C. (2006). A morphological approach to documenting the domestication of Chenopodium in the Andes. Documenting Domestication: New Genetic and Archaeological Paradigms.

[14] Mlakar S.G., Turinek M., Jakop M., Bavec M., Bavec F. (2009). Nutrition value and use of grain amaranth: Potential future application in bread making. Agricultura.

[15] Cheng A. (2018). Shaping a sustainable food future by rediscovering long-forgotten ancient grains. Plant Sci..

[16] Malleshi N. (1989). Processing of small millets for food and industrial uses. Small Millets in Global Agriculture.

[17] Weber S., Kashyap A. (2016). The vanishing millets of the Indus civilization. Archaeol. Anthropol. Sci..

[18] Di Stefano E., White J., Seney S., Hekmat S., McDowell T., Sumarah M., Reid G. (2017). A novel millet-based probiotic fermented food for the developing world. Nutrients.

[19] Mota C., Santos M., Mauro R., Samman N., Matos A.S., Torres D., Castanheira I. (2016). Protein content and amino acids profile of pseudocereals. Food Chem..

[20] Repo-Carrasco-Valencia R., Peña J., Kallio H., Salminen S. (2009). Dietary fiber and other functional components in two varieties of crude and extruded kiwicha (*Amaranthus caudatus*). J. Cereal Sci..

[21] Stikic R., Glamoclija D., Demin M., Vucelic-Radovic B., Jovanovic Z., Milojkovic-Opsenica D., Jacobsen S.-E., Milovanovic M. (2012). Agronomical and nutritional evaluation of quinoa seeds (*Chenopodium quinoa* Willd.) as an ingredient in bread formulations. J. Cereal Sci..

[22] Ruales J., Nair B.M. (1992). Nutritional quality of the protein in quinoa (*Chenopodium quinoa*, Willd) seeds. Plant Foods Hum. Nutr..

[23] Nimbalkar M.S., Pai S.R., Pawar N.V., Oulkar D., Dixit G.B. (2012). Free amino acid profiling in grain Amaranth using LC–MS/MS. Food Chem..

[24] Pedersen B., Kalinowski L., Eggum B. (1987). The nutritive value of amaranth grain (*Amaranthus caudatus*). Plant Foods Hum. Nutr..

[25] Obilana A.B., Manyasa E. (2002). Millets. Pseudocereals and Less Common Cereals.

[26] Ravindran G. (1992). Seed protein of millets: Amino acid composition, proteinase inhibitors and in-vitro protein digestibility. Food Chem..

[27] Malleshi N., Desikachar H. (1986). Nutritive value of malted millet flours. Plant Foods Hum. Nutr..

[28] Parameswaran K.P., Sadasivam S. (1994). Changes in the carbohydrates and nitrogenous components during germination of proso millet, *Panicum miliaceum*. Plant Foods Hum. Nutr..

[29] Nowak V., Du J., Charrondière U.R. (2016). Assessment of the nutritional composition of quinoa (*Chenopodium quinoa* Willd.). Food Chem..

[30] Segura-Nieto M., Vazquez-Sanchez N., Rubio-Velazquez H., Olguin-Martinez L.E., Rodriguez-Nester C.E., Herrera-Estrella L. (1992). Characterization of amaranth (*Amaranthus hypochondriacus* L.) seed proteins. J. Agric. Food Chem..

[31] Guo H., Hao Y., Richel A., Everaert N., Chen Y., Liu M., Yang X., Ren G. (2020). Antihypertensive effect of quinoa protein under simulated gastrointestinal digestion and peptide characterization. J. Sci. Food Agric..

[32] Fan X., Guo H., Teng C., Zhang B., Blecker C., Ren G. (2022). Anti-Colon Cancer Activity of Novel Peptides Isolated from In Vitro Digestion of Quinoa Protein in Caco-2 Cells. Foods.

[33] Vilcacundo R., Miralles B., Carrillo W., Hernández-Ledesma B. (2018). In vitro chemopreventive properties of peptides released from quinoa (*Chenopodium quinoa* Willd.) protein under simulated gastrointestinal digestion. Food Res. Int..

[34] Fillería S.G., Tironi V. (2021). Intracellular antioxidant activity and intestinal absorption of amaranth peptides released using simulated gastrointestinal digestion with Caco-2 TC7 cells. Food Biosci..

[35] Pineli L.d.L.d.O., Botelho R.B., Zandonadi R.P., Solorzano J.L., de Oliveira G.T., Reis C.E.G., Teixeira D.d.S. (2015). Low glycemic index and increased protein content in a novel quinoa milk. LWT-Food Sci. Technol..

[36] Jakubczyk A., Szymanowska U., Karaś M., Złotek U., Kowalczyk D. (2019). Potential anti-inflammatory and lipase inhibitory peptides generated by in vitro gastrointestinal hydrolysis of heat treated millet grains. CyTA-J. Food.

[37] Hu S., Yuan J., Gao J., Wu Y., Meng X., Tong P., Chen H. (2020). Antioxidant and Anti-Inflammatory Potential of Peptides Derived from In Vitro Gastrointestinal Digestion of Germinated and Heat-Treated Foxtail Millet (*Setaria italica*) Proteins. J. Agric. Food Chem..

[38] Sandoval-Sicairos E.S., Milán-Noris A.K., Luna-Vital D.A., Milán-Carrillo J., Montoya-Rodríguez A. (2021). Anti-inflammatory and antioxidant effects of peptides released from germinated amaranth during in vitro simulated gastrointestinal digestion. Food Chem..

[39] Sabbione A.C., Ogutu F.O., Scilingo A., Zhang M., Añón M.C., Mu T.-H. (2019). Antiproliferative effect of amaranth proteins and peptides on HT-29 human colon tumor cell line. Plant Foods Hum. Nutr..

[40] Taniya M., Reshma M., Shanimol P., Krishnan G., Priya S. (2020). Bioactive peptides from amaranth seed protein hydrolysates induced apoptosis and antimigratory effects in breast cancer cells. Food Biosci..

[41] Vilcacundo R., Martínez-Villaluenga C., Hernández-Ledesma B. (2017). Release of dipeptidyl peptidase IV, α-amylase and α-glucosidase inhibitory peptides from quinoa (*Chenopodium quinoa* Willd.) during in vitro simulated gastrointestinal digestion. J. Funct. Foods.

[42] Agrawal H., Joshi R., Gupta M. (2019). Purification, identification and characterization of two novel antioxidant peptides from finger millet (*Eleusine coracana*) protein hydrolysate. Food Res. Int..

[43] Zhang B., Xu Y., Zhao C., Zhang Y., Lv H., Ji X.-M., Wang J., Pang W., Wang X., Wang S. (2022). Protective effects of bioactive peptides in foxtail millet protein hydrolysates against experimental colitis in mice. Food Funct..

[44] Haas R., Schnepps A., Pichler A., Meixner O. (2019). Cow milk versus plant-based milk substitutes: A comparison of product image and motivational structure of consumption. Sustainability.

[45] Manassero C.A., Añón M.C., Speroni F. (2020). Development of a high protein beverage based on amaranth. Plant Foods Hum. Nutr..

[46] Mäkinen O.E., Uniacke-Lowe T., O’Mahony J.A., Arendt E.K. (2015). Physicochemical and acid gelation properties of commercial UHT-treated plant-based milk substitutes and lactose free bovine milk. Food Chem..

[47] Huang K., Zhang S., Guan X., Li C., Li S., Liu Y., Shi J. (2021). Effect of the oat β-glucan on the development of functional quinoa (*Chenopodium quinoa* wild) milk. Food Chem..

[48] Bembem K., Agrahar-Murugkar D. (2020). Development of millet based ready-to-drink beverage for geriatric population. J. Food Sci. Technol..

[49] Luana N., Rossana C., Curiel J.A., Kaisa P., Marco G., Rizzello C.G. (2014). Manufacture and characterization of a yogurt-like beverage made with oat flakes fermented by selected lactic acid bacteria. Int. J. Food Microbiol..

[50] Lorusso A., Coda R., Montemurro M., Rizzello C.G. (2018). Use of selected lactic acid bacteria and quinoa flour for manufacturing novel yogurt-like beverages. Foods.

[51] Balakrishnan G., Agrawal R. (2014). Antioxidant activity and fatty acid profile of fermented milk prepared by *Pediococcus pentosaceus*. J. Food Sci. Technol..

[52] Adebiyi J., Obadina A., Adebo O., Kayitesi E. (2018). Fermented and malted millet products in Africa: Expedition from traditional/ethnic foods to industrial value-added products. Crit. Rev. Food Sci. Nutr..

[53] Isaac-Bamgboye F.J., Edema M.O., Oshundahunsi O. (2019). Nutritional quality, physicohemical properties and sensory evaluation of Amaranth-Kunu produced from fermented grain amaranth (*Amaranthus hybridus*). Annals. Food Sci. Technol..

[54] Ludena Urquizo F.E., García Torres S.M., Tolonen T., Jaakkola M., Pena-Niebuhr M.G., von Wright A., Repo-Carrasco-Valencia R., Korhonen H., Plumed-Ferrer C. (2017). Development of a fermented quinoa-based beverage. Food Sci. Nutr..

[55] Ziarno M., Zaręba D., Henn E., Margas E., Nowak M. (2019). Properties of non-dairy gluten-free millet-based fermented beverages developed with yoghurt cultures. J. Food Nutr. Res..

[56] Jnawali P., Kumar V., Tanwar B. (2016). Celiac disease: Overview and considerations for development of gluten-free foods. Food Sci. Hum. Wellness.

[57] Di Cairano M., Condelli N., Caruso M.C., Marti A., Cela N., Galgano F. (2020). Functional properties and predicted glycemic index of gluten free cereal, pseudocereal and legume flours. LWT-Food Sci. Technol..

[58] Moss R., McSweeney M.B. (2021). Effect of quinoa, chia and millet addition on consumer acceptability of gluten-free bread. Int. J. Food Sci. Technol..

[59] Drub T.F., dos Santos F.G., Centeno A.C.L.S., Capriles V.D. (2021). Sorghum, millet and pseudocereals as ingredients for gluten-free whole-grain yeast rolls. Int. J. Gastron. Food Sci..

[60] Sarabhai S., Tamilselvan T., Prabhasankar P. (2021). Role of enzymes for improvement in gluten-free foxtail millet bread: It’s effect on quality, textural, rheological and pasting properties. LWT-Food Sci. Technol..

[61] Nami Y., Gharekhani M., Aalami M., Hejazi M.A. (2019). Lactobacillus-fermented sourdoughs improve the quality of gluten-free bread made from pearl millet flour. J. Food Sci. Technol..

[62] Jan K.N., Panesar P., Singh S. (2018). Optimization of antioxidant activity, textural and sensory characteristics of gluten-free cookies made from whole indian quinoa flour. LWT-Food Sci. Technol..

[63] Sharma S., Saxena D.C., Riar C.S. (2016). Nutritional, sensory and in-vitro antioxidant characteristics of gluten free cookies prepared from flour blends of minor millets. J. Cereal Sci..

[64] Romero H.M., Santra D., Rose D., Zhang Y. (2017). Dough rheological properties and texture of gluten-free pasta based on proso millet flour. J. Cereal Sci..

[65] Chauhan A., Saxena D., Singh S. (2017). Effect of hydrocolloids on microstructure, texture and quality characteristics of gluten-free pasta. J. Food Meas. Charact..

[66] Rudra S.G., Anand V., Kaur C., Bhooshan N., Bhardwaj R. (2020). Hydrothermal Treatment to Improve Processing Characteristics of Flour for Gluten-Free Pasta. Starch Stärke.

[67] Felix M., Camacho-Ocaña Z., López-Castejón M.L., Ruiz-Domínguez M. (2021). Rheological properties of quinoa-based gels. An alternative for vegan diets. Food Hydrocoll..

[68] Das D., Mir N.A., Chandla N.K., Singh S. (2021). Combined effect of pH treatment and the extraction pH on the physicochemical, functional and rheological characteristics of amaranth (*Amaranthus hypochondriacus*) seed protein isolates. Food Chem..

[69] Ayub M., Castro-Alba V., Lazarte C.E. (2021). Development of an instant-mix probiotic beverage based on fermented quinoa with reduced phytate content. J. Funct. Foods.

[70] Gamel T.H., Linssen J.P., Mesallam A.S., Damir A.A., Shekib L.A. (2006). Seed treatments affect functional and antinutritional properties of amaranth flours. J. Sci. Food Agric..

[71] Melini V., Melini F. (2021). Functional components and anti-nutritional factors in gluten-free grains: A focus on quinoa seeds. Foods.

[72] Panwar P., Dubey A., Verma A. (2016). Evaluation of nutraceutical and antinutritional properties in barnyard and finger millet varieties grown in Himalayan region. J. Food Sci. Technol..

[73] Ravindran G. (1991). Studies on millets: Proximate composition, mineral composition, and phytate and oxalate contents. Food Chem..

[74] Nascimento A.C., Mota C., Coelho I., Gueifão S., Santos M., Matos A.S., Gimenez A., Lobo M., Samman N., Castanheira I. (2014). Characterisation of nutrient profile of quinoa (*Chenopodium quinoa*), amaranth (*Amaranthus caudatus*), and purple corn (*Zea mays* L.) consumed in the North of Argentina: Proximates, minerals and trace elements. Food Chem..

[75] U.S. Department of Agriculture (2019). Agricultural Research Service. FoodData Central. fdc.nal.usda.gov.

[76] Liang S., Liang K. (2019). Millet grain as a candidate antioxidant food resource: A review. Int. J. Food Prop..

[77] Aditya M., Sen D., Bhattacharjee S. (2020). Amaranth: A reservoir of antioxidant-based phytonutrient for combating degenerative diseases. Stud. Nat. Prod. Chem..

[78] Liu M., Zhu K., Yao Y., Chen Y., Guo H., Ren G., Yang X., Li J. (2020). Antioxidant, anti-inflammatory, and antitumor activities of phenolic compounds from white, red, and black Chenopodium quinoa seed. Cereal Chem..

[79] Balakrishnan G., Schneider R.G. (2020). Quinoa flavonoids and their bioaccessibility during in vitro gastrointestinal digestion. J. Cereal Sci..

[80] Zeyneb H., Pei H., Cao X., Wang Y., Win Y., Gong L. (2021). In vitro study of the effect of quinoa and quinoa polysaccharides on human gut microbiota. Food Sci. Nutr..

[81] Dias-Martins A.M., Pessanha K.L.F., Pacheco S., Rodrigues J.A.S., Carvalho C.W.P. (2018). Potential use of pearl millet (*Pennisetum glaucum* (L.) R. Br.) in Brazil: Food security, processing, health benefits and nutritional products. Food Res. Int..

[82] Chávez-Jáuregui R.N., Santos R.D., Macedo A., Chacra A.P.M., Martinez T.L., Arêas J.A.G. (2010). Effects of defatted amaranth (*Amaranthus caudatus* L.) snacks on lipid metabolism of patients with moderate hypercholesterolemia. Food Sci. Technol..

[83] Navarro-Perez D., Radcliffe J., Tierney A., Jois M. (2017). Quinoa seed lowers serum triglycerides in overweight and obese subjects: A dose-response randomized controlled clinical trial. Curr. Dev. Nutr..

[84] Atefi M., Mirzamohammadi S., Darand M., Tarrahi M.J. (2022). Meta-analysis of the effects of quinoa (*Chenopodium quinoa*) interventions on blood lipids. J. Herb. Med..

[85] Pourshahidi L.K., Caballero E., Osses A., Hyland B.W., Ternan N.G., Gill C.I. (2020). Modest improvement in CVD risk markers in older adults following quinoa (*Chenopodium quinoa* Willd.) consumption: A randomized-controlled crossover study with a novel food product. Eur. J. Nutr.

[86] Ren X., Yin R., Hou D., Xue Y., Zhang M., Diao X., Zhang Y., Wu J., Hu J., Hu X. (2018). The glucose-lowering effect of foxtail millet in subjects with impaired glucose tolerance: A self-controlled clinical trial. Nutrients.

[87] Tiwari N., Srivasi S. (2017). Effect of finger millet (*Eleusine coracana*) buns supplementation on serum glucose and serum lipids level in type 2 diabetics. Asian J. Dairy Food Res..

[88] Hou D., Chen J., Ren X., Wang C., Diao X., Hu X., Zhang Y., Shen Q. (2018). A whole foxtail millet diet reduces blood pressure in subjects with mild hypertension. J. Cereal Sci..

[89] Gómez-Cardona E.E., Hernández-Domínguez E.E., Huerta-Ocampo J.Á., Jiménez-Islas H., Díaz-Gois A., Velarde-Salcedo A.J., Barrera-Pacheco A., Goñi-Ochoa A., de la Rosa A.P.B. (2017). Effect of amaranth consumption on diabetes-related biomarkers in patients with diabetes. Diabetes Obes. Metab..

[90] Silva V.O., Gregório M.L., Villafanha D.F., de Godoy M.F. (2018). Effects of Intake of Processed Quinoa Seeds on Lipid Profile in Patients with Coronary Heart Disease. Int. J. Sci..

[91] Kumari D., Chandrasekara A., Athukorale P., Shahidi F. (2020). Finger millet porridges subjected to different processing conditions showed low glycemic index and variable efficacy on plasma antioxidant capacity of healthy adults. Food Prod. Process. Nutr..

[92] Ugare R., Chimmad B., Naik R., Bharati P., Itagi S. (2014). Glycemic index and significance of barnyard millet (*Echinochloa frumentacae*) in type II diabetics. J. Food Sci. Technol..

[93] Vedamanickam R., Anandan P., Bupesh G., Vasanth S. (2020). Study of millet and non-millet diet on diabetics and associated metabolic syndrome. Biomedicine.

